# Level of acceptance and parental perception of silver diamine fluoride treatment in a peruvian school. A cross-sectional study

**DOI:** 10.21142/2523-2754-1401-2026-272

**Published:** 2025-12-28

**Authors:** María Elizabeth Cruz-Flores, Janira Graciela Paco-Armestar, Rosa Josefina Roncal-Espinoza, Alfredo Carlos Manuel Rendón-Alvarado

**Affiliations:** 1 División de Odontopediatría, Universidad Santo Toribio de Mogrovejo. Chiclayo, Perú. mcruz@usat.edu.pe Universidad Católica Santo Toribio de Mogrovejo División de Odontopediatría Universidad Santo Toribio de Mogrovejo Chiclayo Peru mcruz@usat.edu.pe; 2 Universidad Santo Toribio de Mogrovejo. Chiclayo, Perú. pacojanira13@gmail.com Universidad Católica Santo Toribio de Mogrovejo Universidad Santo Toribio de Mogrovejo Chiclayo Peru pacojanira13@gmail.com; 3 División de Odontología Restauradora y Estética, Universidad Santo Toribio de Mogrovejo. Chiclayo, Perú. rroncal@usat.edu.pe Universidad Católica Santo Toribio de Mogrovejo División de Odontología Restauradora y Estética Universidad Santo Toribio de Mogrovejo Chiclayo Peru rroncal@usat.edu.pe; 4 División de Rehabilitación Oral, Universidad Santo Toribio de Mogrovejo. Chiclayo, Perú. alfredoren@gmail.com Universidad Católica Santo Toribio de Mogrovejo División de Rehabilitación Oral Universidad Santo Toribio de Mogrovejo Chiclayo Peru alfredoren@gmail.com

**Keywords:** Silver Diamine Fluoride, Patient Acceptance of Health Care, Perception, Parents, tratamiento con fluoruro, aceptación de la atención de salud, percepción, padres

## Abstract

**Objective:**

To evaluate the level of acceptance and perception of parents regarding treatment with silver diamine fluoride (SDF). Materials and

**Methods:**

: Descriptive, quantitative, cross-sectional, and prospective study conducted with 124 preschool parents, selected through cluster sampling. A validated survey was used, with a Cronbach's alpha of 0.970 for acceptability and 0.952 for perception. Chi-square was applied for associations (p < 0.05).

**Results:**

65.32% of parents did not accept the application of SDF on anterior teeth, and 66.13% on posterior teeth. However, overall perception was favorable for anterior teeth (71.77%) and posterior teeth (77.5%). No statistically significant differences were found between anterior and posterior teeth in terms of acceptance or perception. Additionally, education levels showed a significant association with acceptance**.**

**Conclusion:**

Although parent perception was favorable, the acceptability of SDF was low, primarily due to its aesthetic impact. Education levels influenced the acceptance decision.

## RESUMEN

**Objetivo:**

Evaluar el nivel de aceptación y percepción de los padres frente al tratamiento con flúor diamino de plata (FDP).

**Materiales y métodos:**

Estudio descriptivo, cuantitativo, transversal y prospectivo realizado en 124 padres de preescolares, seleccionados por muestreo por conglomerado. Se utilizó una encuesta validada con un alfa de Cronbach de 0,970 para aceptabilidad y 0,952 de percepción. Se aplicó chi cuadrado para las asociaciones (p < 0,05).

**Resultados:**

El 65,32% de padres no aceptó la aplicación de FDP en dientes anteriores y el 66,13%, en posteriores. Sin embargo, la percepción fue favorable en dientes anteriores (71,77%) y posteriores (77,5%). No se encontró diferencias estadísticamente significativas entre dientes anteriores y posteriores en cuanto a aceptación ni percepción. Además, el nivel educativo mostró asociación significativa con la aceptación.

**Conclusión:**

Aunque la percepción de los padres fue favorable, la aceptabilidad del FDP fue baja, especialmente por su impacto estético. El nivel educativo influyó en la decisión de aceptación.

## INTRODUCTION

Silver diamine fluoride (SDF) is a colorless alkaline substance that was approved by the U.S. Food and Drug Administration (FDA) ^1^. It contains fluoride and silver, whose combination provides remineralization and antibacterial properties, respectively ^2^. The application of SDF is considered a non-invasive, cost-effective, fast, and simple technique, making it highly beneficial for the treatment of fearful children and adults, as well as patients with special needs, salivary dysfunction, and those whose treatment is challenged by medical or behavioral issues ^3^. This product should not be applied to individuals who are allergic to any of its components, pregnant patients, or those with ulcerative gingivitis and stomatitis; it is also contraindicated in patients with pulp inflammation or caries involving the pulp ^4^.

One of the concerns that parents have regarding the use of SDF is the dental aesthetics of their children, as the substance causes irreversible black stains on the teeth. Additionally, it can cause lesions on the oral mucosa due to the oxidizing effect of the product ^5^. A study conducted in Saudi Arabia showed that 87.3% of parents were satisfied with the benefits offered by SDF, while 23.8% were dissatisfied due to the poor aesthetic appearance of the teeth, as they were concerned and feared that the stains would affect their children's self-esteem ^6^. Similar results were found in Hong Kong, where 20% of parents rejected this therapy due to the displeasing black coloration on the anterior teeth. This was likely due to a lack of knowledge about this type of fluoride, as they believed that their children's cavities would worsen because of the stains, and that these would negatively influence their children's appearance and self-image ^7^.

Given the global issue of parental rejection of the use of this product in children, it highlights the limited knowledge about its positive and negative effects, making it necessary to understand the local idiosyncrasy. Currently, SDF is applied as part of preventive interventions in oral health programs in Peru or as a non-invasive treatment in private dental offices.

There are still controversies regarding the acceptance of SDF therapy, as parents have not fully embraced the use of this material due to the dental discoloration it causes ^8^. Additionally, some parents lack knowledge about the benefits and certain disadvantages, such as the dental pigmentation that comes with topical applications of silver diamine fluoride ^9^, resulting in diminished acceptability of this preventive therapy due to the temporary alteration in dental aesthetics ^7^.

This research aims to provide data that will help the dental community raise awareness among parents through educational talks in schools and health centers about the use of SDF, thus encouraging a larger population to accept this material as a preventive treatment. The World Health Organization (WHO) recommends the annual or semi-annual application of this fluoride to halt the progression of caries ^10^. Furthermore, the population in various regions of world will benefit from this and experience an improvement in their quality of life related to oral health.

Therefore, the purpose of this study was to assess the level of acceptance and overall perception of parents regarding treatment with silver diamine fluoride.

## MATERIAL AND METHODS

### Study design and ethical aspects

This research is descriptive, cross-sectional, quantitative, observational, and prospective. The study followed the guidelines of the Helsinki Declaration, did not cause harm to the health or life of the participants, and all were treated equally, without distinction or preference. The participants' rights were respected, and they received a physical informational sheet explaining the purpose of the study. They had to give their consent to either accept or decline participation in the research.

As the first step, approval was requested from the Research Ethics Committee of the Faculty of Medicine at the Universidad Católica Santo Toribio de Mogrovejo, which was granted through Resolution No. 162-2024-USAT-FMED. 

### Sample

The sample size was determined using the formula for proportion estimation, resulting in a sample size of 124 participants, with an adjustment for 10% potential loss. For participant selection (sampling), cluster sampling was used, and the following selection criteria were applied: parent or legal guardian capable of reading and understanding Spanish, over the age of majority, of either sex, with one or more preschool children enrolled at the "IEI N° 121 - Nuestra Señora del Pilar" Educational Institution during the year 2024.

### Data collection

The data were collected using a questionnaire, which was divided into four parts. The first part consisted of a script where parents were informed about the new treatment (SDF) for dental caries. The next section was dedicated to sociodemographic data while ensuring the confidentiality of the participants. The third part included two questions to assess the acceptance of the new product on anterior and posterior teeth with dental caries, for which photographs proposed by the author of the survey were used. The responses were: unacceptable, almost unacceptable, undecided, almost acceptable, and acceptable. Final values were categorized as: unacceptable (1-3) and acceptable (4-5).

Lastly, six questions were included to measure the parents' perception of using SDF on anterior teeth and six questions for posterior teeth, with responses categorized as strongly disagree, disagree, undecided, agree, and strongly agree, with scores ranging from 1 to 5. Final values were classified as: unfavorable (6-15) and favorable (16-30).

### Analysis Plan

The data collection was processed using SPSS (Statistical Package for the Social Sciences, IBM®) Version 27.0, which was used to generate tables according to the established objectives. Additionally, Chi-square tests and p-values were employed to determine the relationship between the variables.

## RESULTS

The study included 124 parents from an initial educational institution in the city of Chiclayo, Peru, of which the majority were women (87.90%) with a predominant age range of 32 to 45 years (58.87%) and higher education (Post-secondary education) (58.06%).

Regarding the parents' acceptance of treatment with SDF, 65.32% of parents considered its use on anterior teeth unacceptable, while a similar result was obtained for posterior teeth, with 66.13%. Furthermore, as shown in Tables 1 and 2, parents with a higher education level showed a higher percentage of rejection toward SDF (61.73% for anterior teeth and 60.98% for posterior teeth). When performing the inferential analysis, a statistically significant association was found between the education level and acceptability on anterior teeth (p = 0.043; X2 = 6.283) (Table 1), and on posterior teeth (p = 0.044; X2 = 6.225) (Table 2). However, no significant relationship was found with the variables of sex or age.

**Table 1:** Level of Parent Acceptance Regarding Silver Diamine Fluoride Treatment on Anterior Teeth According to Sex, Age, and Education Level

Variable	Values	Acceptability on Anterior Teeth				X^2^	p
		Acceptable		Not Acceptable			
		n	%	n	%		
Age	18-31	19	44.19	20	24.69	5.777	0.056
	32-45	22	51.16	51	62.96		
	46-60	2	4.65	10	12.35		
	Total	43	100.00	81	100.00		
Sex	Male	4	9.30	11	13.58	0.483	0.487
	Female	39	90.70	70	86.42		
	Total	43	100.00	81	100.00		
Education Level	Primary	3	6.98	0	-	6.283	0.043*
	Secondary	18	41.86	31	38.27		
	Higher Education	22	51.16	50	61.73		
	Total	43	100.00	81	100.00		

X^2^= Chi-square

*Significant

**Table 2:** Level of Parent Acceptance Regarding Silver Diamine Fluoride Treatment on Posterior Teeth According to Sex, Age, and Education Level

Variable	Values	Acceptability on Posterior Teeth				X^2^	p
		Acceptable		Not Acceptable			
		n	%	n	%		
Age	18-31	17	40.48	22	26.82	2.521	0.284
	32-45	22	52.38	51	62.20		
	46-60	3	7.14	9	10.98		
	Total	42	100.00	82	100.00		
Sex	Male	6	14.29	9	10.98	0.286	0.593
	Female	36	85.71	73	89.02		
	Total	42	100.00	82	100.00		
Education Level	Primary	3	7.14	0	-	6.225	0.044*
	Secondary	17	40.48	32	39.02		
	Higher Education	22	52.38	50	60.98		
	Total	42	100.00	82	100.00		

X^2^= Chi-square

*Significant

Regarding the parent perception of treatment with SDF, it was found that 71.77% expressed a favorable perception of using SDF on anterior teeth and 71.77% for posterior teeth. However, the statistical analysis showed no significant differences between perception and the variables of sex, age, and education level, both for anterior teeth (p = 0.353, p = 0.639, p = 0.486, respectively) (Table 3) and for posterior teeth (p = 0.0493, p = 0.874, and p = 0.480, respectively) (Table 4).

**Table 3:** Level of Parent Perception Regarding Silver Diamine Fluoride Treatment on Anterior Teeth According to Sex, Age, and Education Level

Variable	Values	Perception on Anterior Teeth				X^2^	P
		Favorable		Unfavorable			
		n	%	n	%		
Age	18-31	30	33.70	9	25.71	2.081	0.353
	32-45	49	55.06	24	68.57		
	46-60	10	11.24	2	5.72		
	Total	89	100.00	35	100.00		
Sex	Male	10	11.24	5	14.29	0.220	0.639
	Female	79	88.76	30	85.71		
	Total	89	100.00	35	100.00		
Education Level	Primary	3	3.37	0	-	1.442	0.486
	Secondary	36	40.45	13	37.14		
	Higher Education	50	56.18	22	62.86		
	Total	89	100.00	35	100.00		

X^2^= Chi-square

**Table 4:** Level of Parental Perception Regarding Silver Diamine Fluoride Treatment on Posterior Teeth According to Sex, Age, and Education Level

Variable	Values	Perception of Posterior Teeth				X^2^	P
		Favorable		Unfavorable			
		n	%	n	%		
Age	18-31	31	33.33	8	25.82	1.416	0.493
	32-45	52	55.92	21	67.74		
	46-60	10	10.75	2	6.45		
	Total	93	100.00	31	100.00		
Sex	Male	11	11.83	4	12.90	0.025	0.874
	Female	82	88.17	27	87.10		
	Total	93	100.00	31	100.00		
Education Level	Primary	3	3.23	0	-	1.466	0.480
	Secondary	38	40.86	11	35.48		
	Higher Education	52	55.91	20	64.52		
	Total	93	100.00	31	100.00		

X^2^= Chi-square

Finally, when comparing the acceptance and perception between anterior and posterior teeth (Table 5), no statistically significant differences were found (p > 0.05).

**Table 5:** Level of Parent Acceptance and Parent Perception Regarding Silver Diamine Fluoride Treatment

Variables		Anterior Teeth		Posterior Teeth		X2	P
		N	%	n	%		
Acceptance	Not Acceptable	81	65.32	82	66.13	0.000	1.000
	Acceptable	43	34.68	42	33.87		
	Total	124	100.00	124	100.00		
Perception	Favorable	89	71.77	93	75.00	0.186	0.666
	Unfavorable	35	28.23	31	25.00		
	Total	124	100.00	124	100.00		

X^2^= Chi-square

## DISCUSSION

SDF is an alkaline, colorless solution ^11^. Due to its ease of application, it has emerged as an innovative, non-invasive therapy for the treatment and prevention of dental caries, especially in children who may find conventional treatments challenging ^12^. This material, due to its composition of 24%-27% silver, 7.5%-11% ammonia, and 5%-6% fluoride, provides antimicrobial properties to inhibit the growth of cariogenic pathogens and harmful metabolites that lead to caries progression. Fluoride ions facilitate the remineralization process of the dental structure, while ammonia raises the pH and acts as a stabilizer, thus helping to halt and prevent the progression of dental caries ^5^^,^^13^.

Despite these advantages, the acceptability of SDF remains a controversial issue for parents due to the black pigmentation that occurs after its application on the dental surface, which is considered unaesthetic ^13^. This is reflected in the present study, as we observed that 65.32% and 66.13% of participants would not accept this material as a treatment for anterior and posterior teeth, respectively. This may indicate a lack of knowledge among parents about the benefits of SDF application, as demonstrated in Hong Kong, where around 20% of parents rejected the use of SDF due to concerns about the black coloration appearing on their children's anterior teeth. This seems to be related to a lack of understanding about the treatment, as many parents mistakenly believed that the stains indicated worsening caries and feared that they would affect their children's appearance and self-esteem ^7^. There are clinical studies that support the biannual application of SDF, with a 38% success rate in halting caries progression. However, it has significant disadvantages, including black pigmentation on the teeth, pulp irritation, and irritation of the oral soft tissues. For this reason, other materials such as potassium iodide (KI) are being explored, which, when applied immediately after SDF, improve the aesthetic outcomes. This is due to a reaction with free silver ions on the surface treated with SDF, forming silver iodide, creating a creamy white reaction product. However, some studies indicate that it may not be effective in preventing staining or that it does not provide complete prevention, but rather reduces it ^14^^,^^15^.

Similarly, Bagher et al. ^16^ in their study observed that half of the parents did not accept the treatment, with 43.4% rejecting it due to staining after the application of SDF, and only 1.9% of participants found the staining acceptable. They also showed significantly higher acceptance of SDF treatment on their children's posterior teeth compared to anterior teeth in the dentition. On the other hand, Wajahat et al. ^8^ in their research demonstrated a positive attitude, with 65% of participants accepting the SDF treatment. However, it was discovered that 48.2% of parents were concerned about the aesthetics after treatment with this fluoride, while 20.3% fully accepted the SDF treatment. Furthermore, better acceptance was shown for the treatment of posterior teeth. For the reasons outlined, it is more likely that a dentist would recommend SDF as a treatment option for posterior teeth than for the anterior dentition. This could gain acceptance among caregivers due to its ease of application, greater safety compared to other techniques such as sedation or general anesthesia in uncooperative children, convenience, and cost-effectiveness ^8^. Given the low acceptability of SDF, there is currently ongoing consideration for the development of new materials with strong antibacterial properties to address the staining disadvantage of SDF, including the use of silver nanoparticles. Due to their nanometric size and expansive surface area, they are capable of directly interacting with bacteria, resulting in a bactericidal effect ^14^^,^^15^.

The study by Hanouf et al. ^17^ compared the perception of SDF use to stop dental caries based on the participants' sex, revealing a statistically significant sex difference, with a higher number of women than men expressing strong satisfaction with the material. This indicates that women showed greater satisfaction with the benefits of SDF compared to men. This could be attributed to their involvement in caring for their children and their desire for more convenient restorative solutions. Similarly, Alhabdán et al. ^6^ in their study on knowledge, attitudes, and perceptions of SDF concluded that 22.8% of participants had a neutral opinion about the application of SDF on deciduous anterior teeth, while 33.2% of parents had a favorable perception and attitude toward the treatment with this product on posterior teeth. Additionally, 60.5% of parents agreed with the application of this material due to the lack of cooperation from their young children, requiring the use of other behavioral management techniques.

This study aligns with reporting a higher favorable perception of SDF on posterior teeth, followed by anterior teeth. It also demonstrates that higher education level yielded significant statistical results, indicating that the parents' perception is influenced by this factor. Similarly, Almarwan et al. ^18^ in their research study found that 37% of parents indicated that aesthetics was important to them. Parental acceptance varied depending on the location and type of tooth, with rejection of the application of SDF on permanent teeth ^18^.

Finally, although SDF offers advantages as an easy-to-use, cost-effective, and non-invasive cariostatic treatment, its acceptance is limited by aesthetics, especially on anterior teeth. From a clinical standpoint, it is important to inform parents about the benefits, explain alternatives, and consider the dental location for the application of SDF.

Regarding the study limitations, a cross-sectional design was used with a limited sample, so it could not be considered fully representative and cannot be generalized to other contexts. However, it may lead to future research.

## CONCLUSIONS

This study allowed for the assessment of the level of acceptance and perception of parents regarding treatment with SDF, revealing that while the overall parental perception was favorable, especially due to the material's usefulness in caries control in young children, acceptance was limited due to the aesthetic implications of the treatment, particularly the darkening of the treated dental surfaces. This rejection was more noticeable in the anterior sector. The findings highlight the importance of communicating the benefits of SDF to parents and its implications in relation to a non-invasive dental caries treatment for preschool children, enabling them to make an informed decision about its use as a non-invasive therapeutic alternative in dental consultations.
